# *Puccinia striiformis* f. sp. *tritici* effectors in wheat immune responses

**DOI:** 10.3389/fpls.2022.1012216

**Published:** 2022-11-07

**Authors:** Nan Wu, Ahmet Caglar Ozketen, Yu Cheng, Wanqing Jiang, Xuan Zhou, Xinran Zhao, Yaorong Guan, Zhaoxia Xiang, Mahinur S. Akkaya

**Affiliations:** ^1^ School of Bioengineering, Dalian University of Technology, Dalian, China; ^2^ Department of Chemistry, Middle East Technical University, Ankara, Turkey

**Keywords:** *Puccinia striiformis* f. sp. *tritici* (Pst), effectors, secretome, wheat, yellow (stripe) rust, *Nicotiana benthamiana*, PTI, ETI

## Abstract

The obligate biotrophic fungus *Puccinia striiformis* f. sp. *tritici*, which causes yellow (stripe) rust disease, is among the leading biological agents resulting in tremendous yield losses on global wheat productions per annum. The combatting strategies include, but are not limited to, fungicide applications and the development of resistant cultivars. However, evolutionary pressure drives rapid changes, especially in its “effectorome” repertoire, thus allowing pathogens to evade and breach resistance. The extracellular and intracellular effectors, predominantly secreted proteins, are tactical arsenals aiming for many defense processes of plants. Hence, the identity of the effectors and the molecular mechanisms of the interactions between the effectors and the plant immune system have long been targeted in research. The obligate biotrophic nature of *P. striiformis* f. sp. *tritici* and the challenging nature of its host, the wheat, impede research on this topic. Next-generation sequencing and novel prediction algorithms in bioinformatics, which are accompanied by *in vitro* and *in vivo* validation approaches, offer a speedy pace for the discovery of new effectors and investigations of their biological functions. Here, we briefly review recent findings exploring the roles of *P. striiformis* f. sp. *tritici* effectors together with their cellular/subcellular localizations, host responses, and interactors. The current status and the challenges will be discussed. We hope that the overall work will provide a broader view of where we stand and a reference point to compare and evaluate new findings.

## Introduction

The underlying molecular mechanisms of the complex, dynamic, and multilayer nature of interactions between plants and pathogens are still elusive in many agriculturally crucial plant species and host-specific pathogens, such as *Puccinia striiformis* f. sp. *tritici* (*Pst*), causing yellow rust disease on wheat. Over the years, genetic studies using marker-assisted selection for producing yellow rust-resistant wheat cultivars not only laid the foundation to identify many resistance gene loci (Dracatos et al., 2016) but also allowed map-based cloning of some resistance genes. The advent of genome sequencing and other methods such as MutRenSeq (Steuernagel et al., 2016) and MutChromSeq (Sánchez-Martín et al., 2016) will facilitate the fast cloning of many new yellow rust R (YR) genes. Yellow rust disease is a devastating wheat disease; its destruction will be even more severe in a currently experienced and steadily elevated rapid climate change (Dudney et al., 2021), which further threatens food security. The ability to spread long distances and survival of over-seasoning cause widespread propagation and acceleration of the frequency of genetic variation over time (Jin et al., 2020). Genome-wide sequencing of *Pst* provides comprehensive data analysis as an eximious predictive tool and helps to understand the population characteristics that mirror genomic differences of races in different regions and allows diagnostics and surveillance in hot spot areas more concretely (Hubbard et al., 2015; Bueno-Sancho et al., 2017; Radhakrishnan et al., 2019). Additionally, the sequence information of many *Pst* races provides data to identify the pathogenic factors to excavate plant immunity and interactions between plants and pathogens.

The relationship between plants and pathogenic microorganisms is a co-evolutionary process. To cope with the invasion of complex pathogens, higher plants make use of a large number of cell surface and intracellular immune receptors to sense a variety of pathogenic signals and develop a complete immune system. On the other hand, pathogens need to overcome the host’s immune system for its differentiation and development to further their propagation. Plants have specific receptors to sense pathogens that are called pattern recognition receptors (PRRs), e.g., flagellin epitope (flg22) is a pathogen-associated molecular pattern (PAMP), which is recognized by a specific PRR named flagellin-sensing 2 (FLS2) (Boller and Felix, 2009; Zipfel, 2009). The detection of PAMPs by PRRs stimulates immunity, which is called PAMP-triggered immunity (PTI) (Jones and Dangl, 2006). Once PTI is activated, a series of signaling takes place to counter pathogen attacks, i.e., stomatal closure to prevent pathogen invasion, cell wall thickening and lignification or callose deposition, ion fluxes and oxidative burst; release of reactive oxygen species (ROS), synthesis and release of defense-related hormones; ethylene and salicylic acid (Zipfel and Robatzek, 2010). The PTI response is a massive, repelling, and intimidating shield against the pathogen nuisance. However, successful pathogens can evade, suppress, or manipulate the PTI phenomenon with the aid of specific and small proteinaceous compounds called effectors. Phytopathogen effectors could inhibit plant defense-related enzymes, block or seize recognition of PAMPs, and jam the signaling system. Plants developed another defense strategy to protect themselves against the effectors, which is called effector-triggered immunity (ETI) (Jones and Dangl, 2006). In ETI, effectors are sensed directly or indirectly by the cytoplasmic receptor proteins sharing common features such as N-terminal coiled-coil (CC) or Toll/interleukin-1 receptor (TIR) domains, nucleotide-binding (NB) domain, and leucine-rich region (LRR) (Kobe and Kajava, 2001; Bej et al., 2014) in the carboxyl-terminal. They are referred to as NB and oligomerization domain (NOD)-like receptors (NLRs), depending on the type of the domains, CC or TIR, then they are called CC NLRs (CNLs) and TIR-type sensor NLRs (TNLs), respectively. These two different domains also determine diverse paths of resistance responses (Feys et al., 2005; Day et al., 2006). The LRR of the receptor recognizes effectors secreted by a pathogen, whereas the NB domain is responsible for ATP/ADP binding (Jones and Dangl, 2006). Upon effector and ATP binding, NLR becomes activated (Wang et al., 2019a). Recently in a significant work, the three-dimensional (3D) structure of the active form of HOPZ-ACTIVATED RESISTANCE 1 (ZAR1) was reported (Wang et al., 2019a). It indicated that ZAR1 forms a pentameric complex through CC domains of the monomers on the plasma membrane (PM). The complex punctuates PM that works as a calcium channel. ZAR1 (HOPZ-ACTIVATED RESISTANCE 1) channel activity was shown to be required for triggering ROS prior to programmed cell death (PCD) (Wang et al., 2019b; Bi et al., 2021). Thus, terminating the pathogen invasion and achieving ETI and resistance. The discovery deepens our understanding of PCD at the molecular level for resistance that is activated by a host-specific effector; such effectors are specifically called avirulence factors (Avrs). Until the pathogen averts the recognition of its Avr effector by mutation, elimination, or evolving another effector that inhibits the inspection by NLRs, the cognate R protein maintains disease incompatibility of the plant or the resistance. Once Avr can no longer be sensed, the pathogen becomes virulent again, achieving compatibility.

Upon PCD, ETI-stimulated responses occur and lead to an array of secondary events similar to PTI. PTI can be considered as an extensive defense to a broad range of pathogens including non-host and non-adaptive pathogens due to PAMP perception, whereas ETI is an intensive defense against host-specific pathogens. Accumulating evidence suggests that PTI and ETI are indeed intertwined with each other. Some of the components in PTI and ETI are required for both types of immunity (Chang et al., 2022). PTI and ETI increase the effect of immunity synergistically. It is shown that PRRs are involved in PTI, recognizing apoplastic effectors, which are also required for intracellular ETI (Yuan et al., 2021; Ngou et al., 2021). On the other hand, ETI activates/enhances the expression of PTI signaling components (Pruitt et al., 2021; Tian et al., 2021).

Currently, we have a working model of CNL type of NLRs initiating ETI that is deciphered for ZAR1 (HOPZ-ACTIVATED RESISTANCE 1). A clear understanding of the mode of action will also be crucial for elucidating the mechanism of ETI in relation to PTI in wheat yellow rust resistance, provided that a PstAvr is discovered for a cloned cognate wheat YR gene.

## Genomics, transcriptomics, and proteomics

Decline in the cost and advances in next-generation sequencing (NGS) enable generating genomic and transcriptomic data on much more complicated and challenging organisms, e.g., *Pst*. Thus, the ability to make comparisons between different *Pst* isolates and races and attempt to discover virulence and avirulence factors are made possible by sequencing. Similarly, transcript profiles of a pathogen could be monitored at different time intervals and under various environmental conditions during the infection processes—thanks to the accessibility of NGS. Despite a huge *Pst* genome and transcriptome sequence data availability in yellow rust disease, still, few effectors were investigated for their roles in detail.

To present the current state of affairs, **Tables 1** and **2** were organized using data reported in the literature. *Pst* can only be maintained as urediniospores on a living host, hence, the first identification of functional genes in disease was done on urediniospores of *Pst*-78 using a full-length cDNA library (Ling et al., 2007). Gene expression analysis was reported on germinated urediniospores of *Pst*-CYR32 using Expressed Sequence Tags (ESTs) (Zhang et al., 2008). Haustorium-specific genes were identified in the *Pst*-78 cDNA library, and authors defined proteins that are abundant in haustoria and secreted in various infection stages (Yin et al., 2009). A custom-made microarray chip was developed to reveal the expression profile of suspected genes obtained from past reports (Huang et al., 2011). The genome of *Pst*-130 was sequenced using NGS technology (Cantu et al., 2011). Chinese isolate *Pst-*CYR32 (09-001) was sequenced, and the origin of the isolate was analyzed by comparing four *Pst* isolates (*Pst*-CYR23, 104E137A, PK-CDRD, Hu09-2) from different geographical regions (Zheng et al., 2013). *Pst*-78 (2K-041) genome was published along with a detailed comparative analysis between *Pgt* and *Ptt* (Cuomo et al., 2017). Broad Institute released genome sequences of *Pst*-78 (2K-041), *Pst*-1 (3-5-79), *Pst*-127 (08-220), and *Pst*CYR-32 (09-001) (Zheng et al., 2013; Cuomo et al., 2017; Xia et al., 2017). In addition, genome sequences of *Puccinia graminis* f. sp. *tritici*, *Pgt* (CRL 75-36-700-3), and *Puccinia triticina*, *Ptt* (BBDD), were published as a publicly available reference dataset, which is also useful for comparison studies (http://www.broadinstitute.org/) (Duplessis et al., 2011; Cuomo et al., 2017). The genome sequences of the four races, including *Pst*-87/7, *Pst*-08/21 (two UK races), *Pst*-21, *and Pst*-43 (US races), were reported in a publication by Cantu et al. Additionally, the gene expression data belonging to different time points [6 and 14 days post inoculation (dpi)] of the infection were described as well as haustorium-specific genes Cantu et al. (2013). Hubbard et al. (2015) surveyed *Pst* isolates collected from the UK fields of the United Kingdom in 2013. The authors investigated the evolutionary resemblance of harvested *Pst* isolates to the historical ones (14 UK and seven French isolates) and six additional isolates through whole-genome sequencing (WGS). It was discovered that the field isolates were not related to old isolates, but they possibly originated from foreign *Pst* populations. Garnica et al. (2013) reported the sequence data generated from both haustoria and germinated urediniospores of an Australian. In a later study, seven new races of *Pst* were sequenced using NGS and combined with seven older published genomes. A total of 14 races of *Pst* were subjected to correlation analyses in an attempt to predict Avr candidates (Xia et al., 2017). From the Indian subcontinent for the first time, Kiran et al. (2017) adopted NGS to sequence the genomes of *P. striiformis* pathotypes (46S 119, 31, and K). Eighty-one percent of the total annotated genes were successfully identified, and extracellularly secreted proteins were found to be very conserved in the three pathotypes.

**Table 1:** Genome sequences of *Pst*-races.

Isolates/Races	Origin	References	NCBI Bioprojects
PST-78, PST-1, PST-127, PST-CYR-32	US	Cantu et al., 2011	PRJNA60743
Pst-130	US	Cantu et al., 2011	PRJNA51241
104E137A	Australia	Schwessinger et al., 2018	PRJNA396589
Pst-CY32	China	Zheng et al., 2013	PRJNA176877
PST-87/7	UK	Cantu et al., 2013	PRJNA181962
PST-08/21			PRJNA181960
Pst-21			PRJNA181959
Pst-43			PRJNA181957
PST-12/86, PST-12/83, PST-11/13, PST-11/128, PST-11/08	UK	Hubbard et al., 2015	PRJNA257181
PST-78/66	US		
Pst-78	US	Cuomo et al., 2017	PRJNA41279
11-281	US	Xia et al., 2017	PRJNA354804
Pst-127			
12-248			
12-346			
12-368			
PK08-2	Pakistan		
841541:430	Australia		
*P.str*31, *P.str*K, *P.str*46S119	India	Kiran et al., 2017	PRJNA277552 PRJNA277553 PRJNA277554
*P. striiformis* Kranich race isolate 14/106	UK	Bueno-Sancho et al., 2017	PRJEB15280
93-210	US	Xia et al., 2018	PRJNA422914
38S102	India	Aggarwal et al., 2018	PRJNA344021
30 EMS mutagenesis of *Pst* 11-281/ PSTv-18	US	Li et al., 2020	PRJNA587768
PstS0, PstS7	Europe	Schwessinger et al., 2020	PRJNA588102
12-368 (AvYr44-AvYr7-AvYr43-AvYrExp2 cluster)	US	Xia et al., 2020	PRJNA599033
PstS0, PstS1, PstS10 and PstS13	Australia	Ding et al., 2021	PRJNA704774

**Table 2:** Transcriptome, microarray, and proteome studies.

Transcriptome sequence and microarray analysis			
Isolate/Race	Strategy	Sample	References
PST-78	Full-length cDNA library and cDNA clone sequencing	Urediniospores	Ling et al., 2007
PST-78	Wheat GeneChip	Inoculated flag leaves of *Yr39* (resistant) and *yr39* (susceptible) genotypes at 6, 12, 24, 48 and 96 hpi	Coram et al., 2008a
PST-100	Wheat GeneChip	Inoculated and mock-inoculated *Yr5* (resistant) and *yr5* (susceptible) isolines at 6, 12, 24 and 48 hpi	Coram et al., 2008b
CY32	cDNA library construction and EST sequencing	Germinated urediniospores	Zhang et al., 2008
CY31	cDNA library construction and clone sequencing	3, 5 and 8 dpi infected wheat seedlings of genotype Suwon 11	Ma et al., 2009
CY31	cDNA-AFLP	Inoculated and mock inoculated leaves of wheat genotype Suwon11 respectively sampled at 6, 12, 18, 24, 36, 48, 72, 96, 120, 144 and 168 hpi	Wang et al., 2009
PST-78	Construction and sequencing of a haustorial cDNA library	Haustoria isolated from heavily infected wheat leaves of Avocet ‘S’ carrying the Yr8 resistance gene at 8 dpi.	Yin et al., 2009
169E136, 232E137	Microarray	0, 6, 12, 24, 48, 72 hpi inoculated wheat seedlings of Avocet S and Avocet*6/*Yr1*	Bozkurt et al., 2010
CYR23	cDNA-AFLP	Inoculated leaves of wheat genotype Suwon11 harvested at 6, 12, 18, 24, 36, 48, 72, 96, 120, 144 and 168 hpi	Wang et al., 2010
CYR23	cDNA library construction and sequencing	24, 48, 72 hpi infected seedlings of wheat genotype Suwon11	Yu et al., 2010
PST-78	Microarray	12, 24 and 48 hpi, 7 and 14 dpi inoculated wheat of AvSYr5NIL and germinated urediniospores	Huang et al., 2011
CY32	SSH-cDNA library	0, 24, 48, 72, 96 hpi and 7, 10, 13 dpi inoculated wheat seedlings of Shaanmai139	Zhang et al., 2011
PST-08/21	RNA-seq or transcriptome sequencing	6 and 14 dpi infected wheat seedlings of cv Avocet ‘S’ and haustoria isolated from infected leaf at 7dpi	Cantu et al., 2013
PST-78	Microarray	24 and 48 hpi inoculated wheat leaves of AvSYr5NIL and AvSYr39NIL	Chen et al., 2013
104E137A	RNA-seq or transcriptome sequencing	Germinated urediniospores and haustoria	Garnica et al., 2013
CYR32	SSH cDNA library	12, 24, 48 hpi inoculated adult plants of wheat cv Xingzi 9104	Huang et al., 2013
CYR32	GeneChip microarray	0, 12, 36 hpi inoculated wheat seedlings of 92R137 (resistant), R236 (resistant) and Yangmai158 (susceptible)	Jiang et al., 2013
CYR31	EST library construction and sequencing	0, 1, 2, 3 dpi inoculated seedlings of wheat line N9134	Zhang et al., 2014
CYR23, CYR31	Microarray (microRNA)	0, 12, 24, 48, 72, 120 hpi inoculated wheat cv Suwon11	Feng et al., 2015a
CYR32	Microarray (microRNA)	0, 24, 48, 120 hpi inoculated wheat cv Xingzi9104	Feng et al., 2015b
Mixture of *Pst* (UK field isolates in 2013)	RNA-seq or transcriptome sequencing	PST-infected wheat and triticale collected directly from the field	Hubbard et al., 2015
PST-78/66, PST-12/86, PST-12/83, PST-11/13, PST-11/128, PST-11/08		Infected leaves of susceptible wheat variety Vuka	
PST-87/66	RNA-seq	0, 1, 2, 3, 5, 7, 9, 11 dpi infected seedlings of the susceptible variety Vuka and 0, 1, 2, 3, and 5 dpi inoculated seedlings of the resistant Avocet-*Yr5* line	Dobon et al., 2016
CYR32	RNA-seq or transcriptome sequencing, DGE library construction and sequencing	Adult plant and seedling of wheat cv Xingzi9104 at 0 hpi without *Pst*. 24, 48, 120 hpi inoculated wheat cv Xingzi 9104 at adult plant and seedling stage.	Hao et al., 2016
CYR31	EST library construction and sequencing	0, 1, 2, 3 dpi inoculated seedlings of wheat line N9134	Zhang et al., 2016
CYR31	RNA-seq or transcriptome sequencing	0, 0.25, 0.5, 1, 1.5, 2, 3, 4, 5, 7, 10, and 13 dpi inoculated leaves of wheat line N9134	Zhang et al., 2019a
*Pst* from the fields of Svalöv, Sweden	RNA-seq or transcriptome sequencing	Leaf materials from resistant and susceptible lines were collected from penultimate leaves pooled from three plants from each breeding line which obtained from the cross (Nimbus/3/SW, 2081221/2/SW2-7/Kranich) of the segregating population at the booting stage	Kushwaha et al., 2020
*Pst* isolates from the fields of Anatolia, Turkey	RNA-seq or transcriptome sequencing	Mock and 10 dpi inoculated wheat seedlings of Avocet-S and Avocet-YR10	Ozketen et al., 2020
CYR32	RNA-seq or transcriptome sequencing	The samples of wheat cv. Xiaoyan6 at 8 dpi (0 h post-temperature, hptt) and 9 dpi (24 hptt) under different temperature treatments: (i) normal temperature (N), (ii) normal-high-normal temperature (NHN), and (iii) high temperature (H)	Tao et al., 2020
CYR31	RNA-seq or transcriptome sequencing	Urediospores, germ tubes and haustoria	Xu et al., 2020a
NA	RNA-seq or transcriptome sequencing	538 *Pst*-infected plant samples collected across 30 countries from 2014 to 2018	Adams et al., 2021*
CYR34	RNA-seq or transcriptome sequencing	1, 3, 7 dpi inoculated seedlings of wheat cv SM126	Wang et al., 2021a
CYR32, V26	RNA-seq or tarnscriptome sequencing	Entire leaf tissue taken from barberry (*Berberis shensiana*) plants at 3 and 4 dpi and from wheat cv MX169 at 1 and 2 dpi	Zhao et al., 2021
**Proteome studies**			
CYR23, CYR32	two-dimensional electrophoresis and MALDI-TOF MS	Mock and 24, 72 hpi inoculated wheat cv Suwon11	Li et al., 2011
*Pst* isolates from Turkiye	ProteomeLab PF2D and nanoLC-ESI-MS/MS	0, 24 hpi inoculated wheat cv Izgi2001	Maytalman et al., 2013
CYR32	two-dimensional electrophoresis and MS/MS	0, 24, 48 hpi inoculated japonica rice cultivar Nipponbare	Zhao et al., 2014
*Pst* isolates from Czech	nanoLC-MALDI-MS	Urediniospores	Beinhauer et al., 2016
*Pst* isolates from Turkiye	Nano LC-ESI-MS/MS	0, 1, 2, 3, 4 dpi infected wheat cv Seri82	Demirci et al., 2016
CYR23	iTRAQ and LC-ESI-MS/MS	0, 12, 24, 48 hpi inoculated wheat cv Su11	Yang et al., 2016
CYR32	iTRAQ and MALDI-TOF/TOF tandem MS	Urediniospores and germinated urediniospores	Zhao et al., 2016
CYR31, CYR32, CYR33	iTRAQ and LC-MS/MS	Urediniospores and UV-B radiation applied urediniospores	Zhao et al., 2018a
CYR31	iTRAQ and LC-ESI-MS/MS	0, 24, 48, 72 hpi inoculated wheat introgression N9134	Zhang et al., 2019b
CYR32 and CYR32-5 and CYR32-61 acquired by UV-B radiation	iTRAQ and Nano LC-MS/MS	Urediniospores and germinated urediniospores	Zhao et al., 2020

*Rust expression browser (http://www.rust-expression.com).

Initial deep sequencing of the genomes of a *Pst* race is always laborious and costly but allows a valuable reference genome sequence for analyses of other races and transcriptome analyses at a selected state. Microarray profiling is still useful, but only a set of genes with known sequences could be monitored and investigated, not the novel ones. Although directly detecting proteins is valuable, proteome analyses generate a narrow range of information because of the low level and sometimes short duration of protein expressions; it is possible to miss key proteins. Nevertheless, a study about the proteome profile of compatible interactions between wheat and *Pst* revealed some of the proteins involved in pathogenesis (Demirci et al., 2016). Another proteome study listed proteins of *Pst* that are active in urediniospores and germ tubes using the isobaric tag for relative and absolute quantitation (iTRAQ) method and qRT-PCR for validation (Zhao et al., 2016). Alterations in the proteome content of urediniospores in response to the application of UV-B radiation were reported for three different Chinese races (CYR31, CYR32, CYR33) to elucidate deviations in virulence mechanisms (Zhao et al., 2018a).

**Figure 1:**
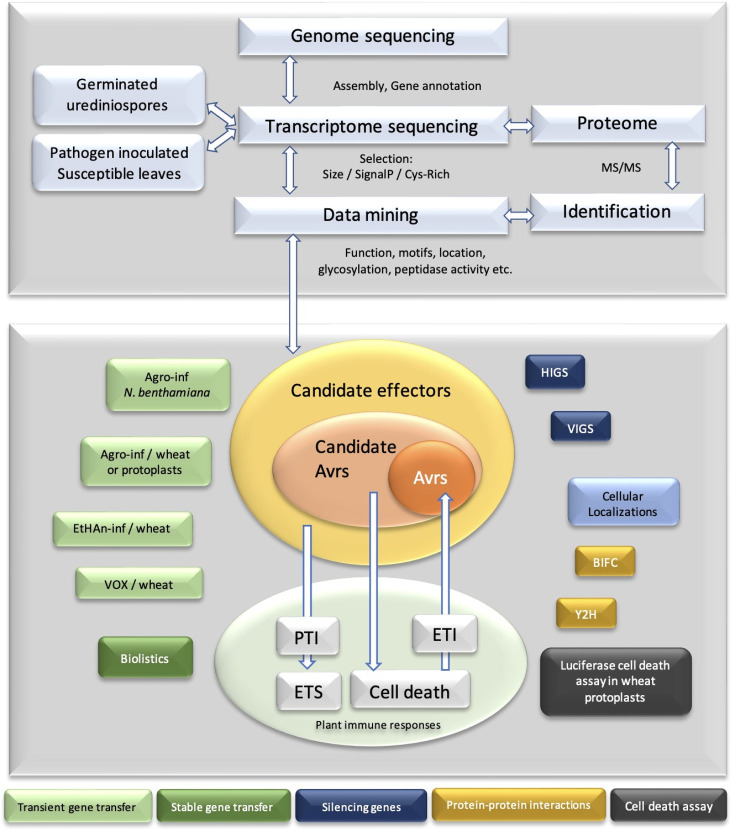
Overview of candidate effector predictions and confirmational analyses.

The effectiveness of omics technologies is obvious in providing bulk data on various races and different phases of the disease or the resistance. Hence, such data are the key resources for mining genes (**Figure 1**).

## Data mining

The pace of effector evolution and the emergence of new races led to the amassed number of suspects in effector biology. The generated data of “omics” related to *Pst* help us to discover, compare, and pinpoint direct and indirect players in pathogenicity and plant resistance mechanisms. Data mining is a popular terminology to explain the studies conducted on big datasets using statistics, predictions, and deep machine learning to evaluate outcomes, to pinpoint crucial subsets of data from bulk collection, and to predict future patterns. Here, data mining is used as a terminology to cover all *in silico* strategies for handling and characterizing bulk data generated from various sequencing strategies to dissect the most relevant information.

Since the datasets obtained through sequencing are quite substantial and testing many numbers of uncovered genes is laborious, time-consuming, and costly, there is a need to pool the most probable sets of candidates so that they can be experimentally tested for function. Consequently, data mining is a useful strategy to narrow down candidate effectors. It uses our prior knowledge about effectors to predict new candidates. For instance, it is known that secreted proteins are important in achieving virulence. Hence, predicting the secreted proteome or “secretome” catalog of a pathogen is an initial step. Of course, the predicted subset may not be fully accurate or can be irrelevant to virulence, but it still concentrates to scan a shorter list of the genes. Each prediction and characterization will increase the success of trials. The first attempt to dissect the secretome was reported on the haustorial cDNA library of *Pst*-78 (Yin et al., 2009). Subsequently, the secretome and effectorome era has begun for many races. Abundant data generated with genomics, transcriptomics, and proteomics were subjected to secretome prediction and characterization by several studies (Cantu et al., 2011; Huang et al., 2011; Cantu et al., 2013; Garnica et al., 2013; Zheng et al., 2013; Demirci et al., 2016; Cuomo et al., 2017; Xia et al., 2017; Xia et al., 2018; Xia et al., 2020; Ozketen et al, 2020). Duplessis et al. (2011) published genome sequences of poplar leaf rust *Melampsora larici-populina* (*Mlp*) and wheat stem rust *Pgt*. Moreover, the group predicted the secretome of the pathogens and small secreted proteins. A pipeline to discover and characterize candidate effector proteins was defined in a hierarchical clustering study using the same data of pathogens, *Mlp* and *Pgt* (Saunders et al., 2012).

The core of secretome prediction is based on two rules: 1) the presence of secretion signal and 2) the absence of transmembrane helices. A protein could be secreted by either classical or non-classical pathways. In the classical pathway, the presence of an N-terminus secretion signal or signal peptide is required for translocation through the endoplasmic reticulum/Golgi-dependent secretory pathway (Nickel, 2003). The non-classical pathway lacks any secretion signal contradictory to a conventional path (Stein et al., 2014). However, secretome prediction is conducted frequently based on classical secretion even though some proteins follow non-classical pathways. The absence of any transmembrane helix is important to rule out any membrane-destined protein. After a secretome is defined, candidate effectors are predicted through certain parameters established on known effectors. Effector proteins are generally short in length. Some apoplastic effectors are rich in their cysteine content to provide stability in the hostile environment of the apoplast. Conserved motifs were also detected in the amino acid sequence of fungal effectors. Most notably, the [FYW]xC motif was identified in a number of candidate effectors of powdery mildew and rust (Godfrey et al., 2010). However, its significance is yet to be clarified. The haustoria provide a handy interface with enough proximity for the effector translocation in a pathogen-dependent or -independent manner. They generally show no homology to known domains except the ones associated with pathogenicity. Effectors could be encoded by genes with long intergenic regions, and they may contain internal repeats. Hence, it becomes possible to set an indefinite number of pipelines for effector mining using different filtering parameters based on known effector functions. A well-accepted pipeline was defined by Saunders et al. (2012) to pinpoint candidate-secreted effector proteins (CSEPs) of fungal pathogens (Duplessis et al., 2011; Saunders et al., 2012). The discovery of each novel effector offers new information for prediction. A list of generated software and databases for effector discovery and characterization is presented in **Supplementary Table S1**. Each one uses different strategies such as sequence similarity, biochemical nature of its composition, and presence of known signals and sequences for diverse sets of tasks including subcellular localization prediction, conserved domain discovery, structure, and function deduction. Among these strategies, machine learning is recently introduced to effector prediction. Algorithms compare and learn experimentally validated sets of positive and negative results in order to forecast a novel protein belonging to an appropriate group. For example, EffectorP is the first reported machine-learning program to predict effectors from other secreted proteins (Sperschneider et al., 2016). EffectorP 2.0, an upgrade for increased accuracy, is released (Sperschneider et al., 2018a). ApoplastP and Localizer are other programs to calculate the subcellular localization of an effector inside/outside the host plant (Sperschneider et al., 2017; Sperschneider et al., 2018b).

Data mining enables filtering amassed numbers of proteins for the probability of relevance, e.g., if the peptidase-like function in virulence is sought after, secretome repertoire can be monitored and sorted for peptidase domains. Undeniably, prediction does not mean that the sorted sets of proteins will always have peptidase function. Subsequently, they should be verified experimentally. Likewise, data mining offers candidate effector functional verifications. Each different pipeline yields a different list of candidate effectors. Hence, each catalog of candidates holds false positives and neglected false negatives. However, the advantages of mitigation of large datasets are greater than the disadvantages. Fungal effectors do not share conserved sequence motifs, sequence similarity, and common features in a broad spectrum. Hence, advances in data mining are essential for effector biology (**Figure 1**).

## Functions and features of effectors

Sequencing of genomes and transcriptomes at various developmental stages following *Pst* infection and gene annotations have largely resolved the grouping of secreted proteins, which can be defined as candidate effectors. Nevertheless, for them to be identified as true pathogen effectors, experimental proof is required to assess their role in PTI and/or ETI, which necessitates labor-intensive experimental verifications of each effector candidate one by one to pinpoint the functions by elucidating the interactions with the host and even the other factors of a pathogen, determining targeted subcellular localizations, hence the mode of action.

**Figure 2:**
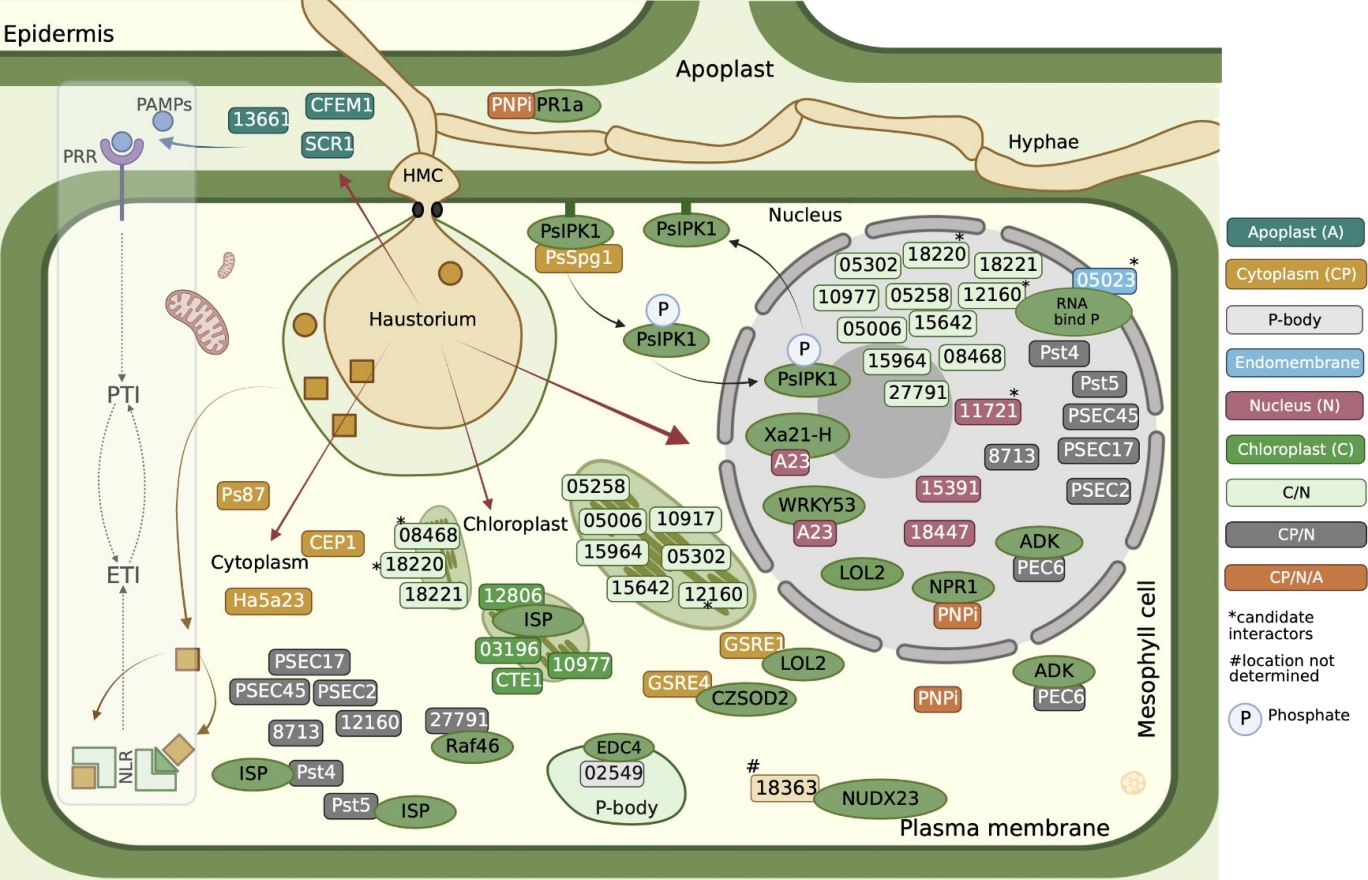
Cellular localization of *Pst* effectors and their known targets. The roles indicated in **Table 3** are related to PTI. Any effector interacting directly or indirectly with NLR that generates ETI is not known. The red arrows point to the locations. Host interactors are shown in green oval boxes. *The candidate interactors were found but not confirmed (**Table 3**). ADK, Adenosine Kinase; CEP1, Candidate Effector Protein 1; CFEM1, Common in Fungal Extracellular Membrane Protein 1; CTE1, Chloroplast Targeting Effector 1; CZSOD2, Copper Zinc Superoxide Dismutase 2; EDC4, ENHANCER OF mRNA DECAPPING PROTEIN 4; ETI, Effector Triggered Immunity; GSRE1, Glycine-serine-rich Effector 1; GSRE4, Glycine-serine-rich Effector 4; ISP, Cytochrome b6–f complex iron–sulfur Subunit; LOL2, LSD1-One-Like-2, LSD1: Lysine Specific Demethylase 1; NLR, Nucleotide Binding Leucine-rich Receptors; NPR1, Non-expresser of PR genes 1; NUDX23, Nudix Hydrolase 23; PAMPs, Pathogen Associated Molecular Pattern; PNPi, Puccinia NPR1 interactor, NPR1: Non-expresser of PR genes 1; PR1a, Pathogenesis-related Protein 1a; PRR, Pattern Recognition Receptor; PsIPK1, Puccinia striiformis-Induced Protein Kinase 1; PsSpg1, Septum-promoting GTP-binding Protein 1; PTI, PAMP Triggered Immunity; Raf46, Raf-like Kinase; RNA bind P, RNA-binding Protein; SCR1, Small Cysteine-rich Protein 1; WRKY53, WRKY Transcription Factor 53; Xa21-H, Homologous to Xa21 in rice.

The following are the studied *Pst* effectors to date (**Table 3**). An effector candidate, *Ps87*, is discovered in the cDNA library of germinated urediniospores of *Pst* CYR32 (Zhang et al., 2008). *Ps87* was reported to bear an RxLR-like motif (Gu et al., 2011). *PEC6* (*Pst* effector candidate 6) was identified to interact with adenine kinase in host cells to suppress PTI by hindering ROS release and obstructing callose deposition (Liu et al., 2016). *PNPi* (Puccinia *NPR1* interactor) has a DPBB-1 (Double-psi beta Barrel Domain-1) domain to interact with *NPR1* (Non-expresser of PR genes 1), which is a central regulator of the defense response gene (Cao et al., 1994; Mou et al., 2003), in the nucleus to jam its interaction with the corresponding transcription factor of defense genes (Wang et al., 2016). Recently, it was shown that *PNPi* targets wheat pathogenesis-related protein TaPR1a in the apoplastic space and can suppress multiple defense responses in wheat plants by targeting different components (Bi et al., 2020). In another study, several *Pst* effector candidates were investigated to pinpoint their interactors inside host cells of *Nicotiana benthamiana* leaves (Petre et al., 2016). Ramachandran et al. (2017) studied nine *Pst* effectors, seven of which were reported to suppress cell death; Shr7 halted the PTI response stimulated by flagellin epitope (flg22) infiltration into *N. benthamiana* leaves (Ramachandran et al., 2017). These researchers assessed the ability of the effectors to suppress a hypersensitive response (HR) with known cytosolic Effector/R combinations, *Cp/Rx*, *ATR13*/RPP13, *Rpt2/RPS2*, and *GPA/RBP1*. *PstHa5a23* is one of the candidate effectors that were identified in the haustorial cDNA library of *Pst-78* (Yin et al., 2009). It was discovered that PstHa5a23 targets the cytoplasm and suppresses cell death triggered by INF1, BAX, MKK1, and NPK1. *PstSCR1* (previously PstHa2a5) is shown to be induced during infection, and it enhances plant immunity, PTI. It elicits severe cell death upon translocation into the apoplastic fluid (Dagvadorj et al., 2017). An effector candidate (*Pst8713*) was shown for its ability to suppress the cell death triggered by *INF1* and *BAX* hampering ROS and callose deposition (Zhao et al., 2018b). Candidate effector *Pst_8713* is found to be highly expressed in early infection, is localized in the host cytoplasm and nucleus, and inhibits PTI-associated callose deposition. The effector *PstGSRE1* acts as an important virulence factor targeting *TaLOL2*, which is a positive regulator of zinc finger protein transcription factor against stripe rust, proposed to block nuclear localization of *TaLOL2* and inhibit host immunity (Qi et al., 2019). *Pst18363* by Yang et al. (2020) was shown to interact with *TaNUX23* and suppress ROS accumulation. *Pst_12806*, which has a predicted chloroplast transit peptide, is translocated into host plant chloroplasts, where it interacts with the Rieske domain of TaISP and attenuates photosynthesis rate, decreases ROS accumulation at the infection sites, and inhibits plant defenses (Xu et al., 2019). *Pst13661* was identified as a polysaccharide deacetylase found to suppress BCL2 Associated X protein (BAX)-induced cell death (Xu et al., 2020a). *PstCTE1* was shown to target chloroplast with an unknown targeting mechanism, since it lacks chloroplast-targeting transit peptide. Red Fluorescent Protein (RFP) blocking the N-terminal of *PstCTE1* does not interfere with its destination to the chloroplast (Andac et al., 2020). *PSEC2* and *PSEC17* both appear in the cytoplasm and chloroplast inhibiting the PTI response of the host (Su et al., 2021). Two stripe rust effector proteins *Pst_4* and *Pst_5* weaken wheat resistance by inhibiting the entry of host ferritin into chloroplasts and interfere with chloroplast-mediated defense by binding to TaISP in the cytoplasm, by which they both inhibit the entry of *TaISP* into chloroplasts (Wang et al., 2021b). *PSTG_10917* localizes in the chloroplast, and it can suppress cell death induced by INFESTIN 1 (IFN1) in an *N. benthamiana* heterologous expression system (Ozketen et al., 2020). The effector *Pst27791* targets wheat Raf-like kinase TaRaf46 to interfere with host immunity including ROS accumulation, expression of Salicylic Acid (SA)-related defense genes *TaPR1/2*, and Mitogen-Activated Protein Kinase (MAPK) activation (Wan et al., 2022). A candidate effector protein *PstCFEM1* facilitates *Pst* infection by suppressing ROS accumulation (Bai et al., 2022). Secreted protein *PstCEP1* (*PSTG_13342*) has the function of suppressing PCD and responds to wheat high-temperature seedling-plant resistance *via* affecting PTI and ETI (Tao et al., 2020). *Pst_A23* acts as a splicing regulator that directly binds the cis-elements of host genes, ultimately resulting in a reduction of the plant defense response (Tang et al., 2022). Recently found *PstGSRE4* (Liu et al., 2022) is a glycine-serine-rich effector that interacts with wheat copper-zinc superoxide dismutase; *TaCZSOD2* inhibits the dismutase activity. Host-induced gene silencing (HIGS) and the overexpression of the effector showed reduced virulence with increased H_2_O_2_ accumulation and increased virulence, respectively. One latest *Pst* effector, *PsSpg1* (Wang et al., 2022), was shown to lack a typical fungal effector signal peptide, which comes as no surprise, since it was detected indirectly as an interactor while investigating the roles of a receptor-like cytoplasmic kinase (*TaPsIPK1*) that was induced by fungus inoculation, and it negatively regulates wheat resistance to yellow rust pathogen. *TaPsIPK*1 appears to be a susceptibility gene. *PsSpg1* was shown to impede the virulence of multiple *Pst* races and promote parasitism *via* enhancing kinase activity and nuclear entry of *TaPsIPK1*. These effectors, except *PsSpg1*, regardless of their subcellular targets, are mostly found as PTI suppressors (**Table 3**, **Figure 2**). In susceptible plants, PTI is overcome by adapted pathogens in which virulence effectors are suppressed to defeat PTI. In the case of *PsSpg1*, it must act in effector-triggered susceptibility.

**Table 3:** The *Pst*-effectors identified to date; functions, features, and host interactors.

Effectors	No. aa	Location	Function / Features / Interactors	References
*Ps87*	85	Cyt	No effect (19, 21, 31)	Gu et al., 2011
*PEC6*	88	Cyt/Nuc	Interactor: ADK1; Function: Suppresses of PTI (1, 2, 3, 10, 11, 12, 16, 24, 26, 27)	Liu et al., 2016
*PNPi*	333	Cyt/Nuc	Interactor: NPR1; Function: Suppresses of defense (4, 7, 12, 16)	Wang et al., 2016
		Apo/Cyt/Nuc	Interactor: TaPR1a; Function: suppress multiple defense responses in wheat plants (4, 7, 12, 13, 16, 24, 28)	Bi et al., 2020
*Pst02549*	297	P bodies	Interactor: EDC4 (7, 14)	Petre et al., 2016
*Pst18220*	110	Chl/Nuc	Candidate interactors: ABC transporter F family member 4, THO complex subunit 2, DNA damage binding protein 1 (7, 14)	
*Pst03196*	206	Chl	(7)	
*Pst05023*	281	EM	Candidate interactor: RNA recognition motif containing protein (7, 14)	
*Pst05258*	256	Chl/Nuc	(7)	
*Pst05006*	201	Chl/Nuc	(7)	
*Pst05302*	160	Chl/Nuc	(7)	
*Pst08468*	206	Chl/Nuc	Candidate interactors: SNF4, SNF4 like protein, SNF1 related protein kinase (7, 14)	
*Pst11721*	250	Nuc	Candidate interactors: Chaperonin, S/T-protein phosphatase 2A, Dihydrodipicolinate reductase 3 (7, 14)	
*Pst18447*	146	Nuc	(7)	
*Pst15391*	256	Nuc	(7)	
*Pst10977*	171	Chl/Nuc	(7)	
*Pst12160*	168	Chl/Nuc	Candidate interactors: Signal recognition particle 54 kDa protein, Oxidoreductase, Ubi 1 (7, 14)	
*Pst15642*	102	Chl/Nuc	(7)	
*Pst18221*	112	Chl/Nuc	(7)	
*Pst15964*	128	Chl/Nuc	(7)	
*Shr1 (Pstg00494)*	199	–	Suppressor of cell death (29)	Ramachandran et al., 2017
*Shr2 (Pstg01062)*	182	–		
*Shr3 (Pstg01724)*	114	–		
*Shr4 (Pstg09266)*	191	–		
*Shr5 (Pstg10812)*	105	–		
*Shr6 (Pstg14250)*	199	–		
*Shr7 (Pstg14695)*	151	–	Suppressor of cell death, PTI and HR (2, 24, 27, 29)	
*PstHa5a23*	108	Cyt	Function: Suppresses of PTI and cell death, virulence (1, 2, 8, 19, 21, 22, 23, 31)	Cheng et al., 2017
*PstSCR1*	116	Apo	Function: Cell death elicitor (7, 20, 25)	Dagvadorj et al., 2017
*Pst8713*	114	Cyt/Nuc	Function: Induced early infection stage, suppresses of PTI and cell death (1, 2, 7, 8, 19, 21, 31)	Zhao et al., 2018b
*Pst18363*	219	–	Interactor: TaNUDX23; Function: Suppresses ROS accumulation (1, 12, 13, 16, 17, 21, 30, 31)	Yang et al., 2020
*PstGSRE1*	290	Cyt	Function: Defeats ROS-induced defense, by inhibiting the transcription factor, TaLOL2 (1, 2, 3, 8, 12, 17, 30, 31)	Qi et al., 2019
*Pst13661*	275	Apo	Function: Polysaccharide deacetylase, suppresses BAX-induced cell death, Interactors: Itself as homopolymer (1, 3, 7, 12, 13, 16, 17, 21, 31)	Xu et al., 2020b
*PstCTE1*	133	Chl	Feature: Novel chloroplast target sequence (7, 8, 9)	Andac et al., 2020
*Pst12806*	146	Chl	Function: Inhibits Bax-induced cell death and Pseudomonas induced cell death, Interactor: TaISP (1, 2, 7, 12, 13, 16, 21, 24, 27, 31)	Xu et al., 2019
*Pst10917*	130	Chl	Function: Inhibits INF1 mediated cell death (7, 19)	Ozketen et al., 2020
*PSEC2*	187	Cyt/Nuc	Function: Inhibition of PTI response (2, 8)	Su et al., 2021
*PSEC17*	257	Cyt/Nuc		
*PSEC45*	230	Cyt/Nuc		
*Pst_4*	152	Cyt/Nuc	Interactor: TaISP, cyt-b6-f complex iron-sulfur subunit, a Chl protein encoded by Nuc gene (1, 2, 4, 12, 13, 16, 21, 31)	Wang et al., 2021b
*Pst_5*	147	Cyt/Nuc		
*Pst27791*	207	Cyt/Nuc	Interactor: TaRaf46; Function: Suppressor of cell death, ROS accumulation and the salicylic acid-dependent defense response, virulence (4, 8, 12, 13, 16, 27, 30, 31)	Wan et al., 2022
*PstCFEM1*	192	Apo	Function: Suppressor of cell death, ROS accumulation and callose deposition, virulence (1, 2, 7, 30, 31)	Bai et al., 2022
*PstCEP1(PSTG_13342)*	243	Cyt	Function: Suppressor of cell death, responding to wheat HTSP resistance via affecting the ETI and PTI, virulence (1, 2, 7, 19, 21, 31)	Tao et al., 2020
*Pst_A23*	181	Nuc	Interactor: cis-element of TaWRKY53 and TaXa21-H; Function: regulate host pre-mRNA splicing, suppresses plant basal defense responses, virulence (2, 4, 7, 8, 15, 21, 24, 26, 31)	Tang et al., 2022
*PsSpg1*	232	Cyt	Interactor: TaPsIPK1; Function: virulence (1, 5, 6, 7, 12, 18)	Wang et al., 2022
*PstGSRE4*	232	Cyt	Interactor: TaCZSOD2; Function: Suppressor of cell death, ROS accumulation and callose deposition, virulence (1, 2, 3, 4, 7, 12, 13, 17, 21, 30, 31)	Liu et al., 2022
*PSTG_01766*	307	Nuc/Cyt/membrane	Interactor: TaPLCP1; Function: Suppress high-temperature seedling resistance (1, 2, 3, 7, 8, 10, 12, 16, 17, 19, 21, 31)	Hu et al., 2022
**Methods**					
Gene transfer into intact wheat			**1)** HIGS *via* BSMV, **2)** *P. fluorescens* (EtHAn)/pEDV6, **3)** Particle bombardment, **4)** Agrobacterium, **5)** *Fusarium graminearum* **6)** BSMV-VOX		
Subcellular localization analysis			**7)** Agrobacterium-mediated transformation of *N. benthamiana*, **8)** Wheat protoplast, **9)** Tobacco protoplast, **10)** Wheat plants, **11)** Transgenic Arabidopsis lines		
Interactors and verification			**12)** Y2H, **13)** CoIP, **14)** CoIP/MS**, 15)** RNA-EMSA, **16)** BIFC, **17)** Pull-down assay, **18)** Split luciferase comp. (SLC) assay		
Identification of PTI involvement			**19)** INF1, **20)** BAK1, **21)** BAX **22)** MKK1, **23)** NPK1, **24)** *P. syringae* DC3000, **25)** *P. infestans*, **26)** *P. fluorences*, **27)** Flg22, **28)** *M. oryzae*, **29)** Cytosolic ‘Effector/R-gene’ combinations: Cp/Rx, ATR13/RPP13, Rpt2/RPS-2, GPA/RBP-1, Pto (Y207D), **30)** Pst322		
Functional validation of the signal peptide			**31)**Yeast invertase secretion assay		

The current understanding of the research of *Pst* protein effector biology is still very limited; other unknown factors, both effectors of pathogen or host, may be facilitating the primary target interactors within the host cell. The activities of effectors most probably are transformed continuously in time and space.

The common methods used to determine the roles of effectors in PTI in the aforementioned studies were conducted in either whole plant (native host or model organism) or protoplasts. The assays contain detection of ROS generation, calcium production, activation of MAPK cascades, induction of defense-related genes, and callose deposition. These methods are well optimized in *N. benthamiana* (Chakravarthy et al., 2009; Nguyen et al., 2010). Also, the consequences of *Pst* effectors, which are overexpressed or silenced, were assessed using the determinants of PTI. For example, PstHa5a23 demonstrated the competence of the effector in suppressing cell death induced by the transient expression of *INF1*, *BAX*, *MKK1*, and *NPK1* on *N. benthamiana* leaves (Cheng et al., 2017). Similarly, the influence of candidate effectors on non-host pathogens was studied using the heterologous system *N. benthamiana*.

In these studies, the experimental methods such as yeast two-hybrid and Co-Immunoprecipitation (Co-IP) and/or pulldown allowed detection of host protein interactors, which are the key determinants of understanding how an effector is mediating its influence. One of the strategies relying on chimeric effector-tagged protein fusions was established to capture interacting partners *in vivo* utilizing FLAG-tag and fluorescent tags (Win et al., 2011; Petre et al., 2016).

Another key determinant to elucidate the function of the effectors is the location of the effector both extracellularly and intracellularly. The location of a protein is meaningful for the evaluation of its biological function. It is expected for a protein to be present in the subcellular location of the interaction site. Similarly, the pathogen effector needs to travel to the location of its target. Exploiting this phenomenon, the biological function or pathogenicity attribution of an effector could be estimated. For instance, an apoplastic effector is more likely to establish favorable conditions by fighting host defensive measures such as defense enzymes. If an effector localizes in the nucleolus of the host cell, it should be expected to be involved in the regulation or interference of transcription. Thereby, investigating the localization site of an effector candidate illuminates its role. It is however imperative to compare and contrast the microscopic analyses with co-infiltration of known cellular markers fused to various fluorescent proteins, green fluorescent protein (GFP), Yellow Flurescent Protein (YFP), mCherry, etc. *Agrobacterium tumefaciens*-compatible plant destination vectors of “Gateway Cloning” methodology were designed previously (Karimi et al., 2002). The pK7FWG2 vector is one of the destination plasmids having a strong 35S promoter site of the cauliflower mosaic virus. Cloning the effector of choice into the plasmid results in effector-GFP fusion at the C-terminal end. The versatility of this system benefitted research related to candidate effector investigation (Liu et al., 2016; Petre et al., 2016; Dagvadorj et al., 2017; Evangelisti et al., 2017).

The studies in **Table 3** uncovered the biological roles of numerous effectors of yellow rust fungi fused to fluorescent protein for their interacting partners and subcellular localizations. In the majority of these studies, *N. benthamiana* is used as a surrogate experimental plant system, which offers a great chance to scrutinize candidate effectors inside plant cells, despite being a non-homologous plant for *Pst* effectors.

## Experimental approaches and limitations

There is a considerable number of methods in effectoromics research including *in silico*, *in vitro*, and *in vivo* approaches that aid in evaluating, dissecting, and filtering these gigantic datasets generated. The most laborious and time-consuming part is to validate each effector candidate for its functions using *in planta* and *in vitro* practices. Currently, a huge number of *Pst* candidate effectors are still awaiting full elucidation of functions in immune responses.

In the case of *Pst* parasite and wheat, to meet the following conditions is extremely challenging to study effectors with high-throughput experimental approaches. Not only is it preferred to study the effectors on their native host but also it is preferred that an effector in question can be overexpressed in the native parasitic organism with peptide tags and/or fused with fluorescent proteins, so recombinantly modified pathogen can be further investigated for functional analyses on the native host. Since *Pst* is an “obligate biotroph” and cannot be cultured *in vitro*, the pathogen cannot be genetically manipulated. The only form of *Pst* that can be obtained is a urediniospore, which can only be germinated on water-agar plates; the effective genetic manipulation of these entities is not currently available. Nevertheless, this limitation is overcome by expressing the *Pst* effectors or their interactors on wheat with various engineered biological entities, which are discussed below.

## *Agrobacterium tumefaciens* mediated transient transformation of wheat by effectors

Inefficient transient transformation in wheat for the high-throughput screening of *Pst* candidate effectors directly on its host slows the research progress. *A. tumefaciens*-mediated transient gene transfer is ineffective in monocots due to compatibility issues (De Cleene and De Ley, 1976). Unlike *N. benthamiana*, simply put, *Agrobacterium* cannot transiently transform intact plant leaves of any wheat cultivar of interest as efficiently as needed. There are reports that when particular *A. tumefaciens* strains, LBA4404 and COR308, are used, the transient gene transfer on some wheat cultivars, e.g., Thatcher (Panwar et al., 2013; Cuomo et al., 2017), is successfully achieved; however, not every cultivar with a particular desired genetic background can be utilized. Thus, various *Agrobacterium* strains should be tested for each wheat variety of interest. It is reported that the recalcitrance of plant species to *Agrobacterium* is “primarily determined by the timing and the intensity at which host defense responses are activated” (Pitzschke, 2013). There are examples in the literature, e.g., for the closest yellow rust parasite, stem rust, where *Agrobacterium* strain AGL1 carrying AvrSr35 (Salcedo et al., 2017) and its cognate Sr35 co-infiltrated into barley successfully showed cell death due to ETI. Other examples are presented in **Table 3**.

## Effector-to-host analyzer for transient transformation in wheat by effector

The other means of transient gene expression is the use of an engineered *Pseudomonas fluorescens* strain. A bacterial delivery system is engineered as Effector-to-Host Analyzer (EtHAn) by harnessing Type 3 Secretion System (T3SS) of *Pseudomonas syringae* pv. *syringae-61* for effector delivery by stably integrating the hrp/hrc region into the genome of *P. fluorescens Pf0-1* (Thomas et al., 2009). The pEDV6 gateway destination vector was constructed by manipulating the N-terminal amino acid sequence of AvrRPS4 for type 3 secretion of any effector of interest cloned into the vector (Sohn et al., 2007). There are concerns raised by the researchers about the lack of reproducibility of the observations with transient gene expression by EtHAn, including us. In our hands, for example, cell death once observed with a *Pst* effector was inconclusive in other trials (unpublished data). We suspect that very minor variations in plant growth conditions are the most probable cause for the observed irreproducibility. The list in **Table 3** presents some of the successful applications.

## Virus-mediated effector overexpression in wheat

A few other alternative means of gene transfer for overexpression in wheat is present, one of which is the utilization of engineered viruses, albeit with other limitations. Barley stripe mosaic virus (BSMV) is effectively used for gene silencing in wheat. However, its use for virus-mediated overexpression (VOX) is only possible for non-native or non-homologous genes; otherwise, instead of overexpression, silencing can occur. There is another limitation even if non-homologous genes are to be overexpressed, which is the requirement of a short insert. BSMV gamma-RNA genome cannot sustain large inserts of full non-homologous genes for overexpression. To overcome such limitations, there are efforts to split the gamma genome of BSMV to maintain stability (Lee et al., 2012). Recently, another virus, a monopartite foxtail mosaic virus (FoMV) was shown to stably overexpress longer proteins, with limitations of up to 600 amino acids (Bouton et al., 2018). There is only one example of VOX of *Pst* effector (232 amino acids) in wheat (Wang et al., 2022) (**Table 3**).

## Effector gene silencing in wheat

Gene silencing strategy is an effective tool to investigate the biological significance of candidate genes by delivering the silencing constructs of antisense RNA. It is widely used as a gene validation tool for observing the change in phenotype. The virus-based elements are engineered for the expression of antisense RNA in host plants as in virus-induced gene silencing (VIGS). BSMV is the most commonly and effectively utilized virus for gene silencing in wheat. It is also used effectively for HIGS of the genes of *Pst*. VIGS is relatively straightforward and trouble-free in functional genomics of plants (Holzberg et al., 2002). In early applications of proviral DNA of BSMV, genomes were transcribed *in vitro* and the RNA generated was used for inoculations. Over a decade, viral RNA generated *in vivo*, *N. benthamiana*, sap containing the virus was used to rub-inoculate wheat seedlings (Yuan et al., 2011). The method reduced the cost and allowed its widespread application. HIGS exploits the ability of double-stranded RNA (dsRNA) or small interfering RNA (siRNA) translocation from a host into a pathogen (Nowara et al., 2010) upon expression of hairpin RNA, antisense, sense, or dsRNA. It is demonstrated that pathogen effector candidates could be subjected to HIGS for assessing the biological function of *Pst* effectors (Yin et al., 2011), since generated dsRNA and/or siRNAs can enter haustoria. Currently, host-induced silencing of the messages of *Pst* effectors is extensively conducted to assess the loss/reduced function of effectors (**Table 3**).

## Effector delivery and expression in wheat protoplasts

The literature is full of use of protoplasts instead of intact plants for many purposes, especially if a robust transient transformation method of a plant is not available, or sometimes protoplasts allow better microscopic detections to assess not only the cellular targets of effectors but also cell death, ROS accumulation, etc. The optimized delivery of plasmids expressing the gene of interest is available for model plants and crops, many of which are referred to in the references of the studies listed in **Table 3**. The method takes advantage of polyethylene glycol (PEG)-mediated delivery of DNA and the use of enzymes for the removal of the cell wall. Nevertheless, the procedure must be optimized in each laboratory. A major shortcoming is the need to isolate protoplasts freshly. One of the best and most recent examples of protoplast microscopic analyses comes from the studies of ZAR1 (HOPZ-ACTIVATED RESISTANCE 1) (Bi et al., 2021), encouraging similar studies to be conducted with wheat protoplasts.

## Conclusive remarks

The biological importance of effectors as virulence and avirulence determinants commenced the new era of effectoromics. Common features of the effectors such as secretion to apoplast or into the plant cell enabled the high-throughput discovery of effectors (**Figure 1**). Any identified unique feature compels further studies to elucidate their functions. Despite the available genome and transcriptome information and ongoing annotations of the genes of *Pst*, to entirely understand the biological and biochemical functions, interactions, cell entrance, organellar targeting, and the mechanism in induction or suppression of plant immunity of hundreds of *Pst* effectors, robust high-throughput functional analysis methods are needed. So far, a heterologous planta system, particularly the model organism *N. benthamiana* with immense examples and generated information, appears as the best system in understanding effector biology.

Most often, also in the case of *Pst*, the search and functional analyses of effectors aim to identify the Avrs. Despite the aforementioned limitations, it is still possible to test avirulence gene candidates by co-infiltrating *N. benthamiana* together with cognate wheat R-genes based on some criteria: 1) provided that the R-gene of interest is a singleton, or 2) if not, heterologous/non-native helper NLRs of *N. benthamiana* can execute an HR, or 3) if the cellular content and/or the genetic background of *N. benthamiana* is conducive for the avirulence factor activating its cognate R-protein. If these requirements are met, detecting cell death in *N. benthamiana* allows the verification of the effector being the avirulence factor of *Pst*. These requirements may limit the identification of many Avrs of *Pst* in a non-host system, *N. benthamiana*.

Currently, the use of wheat protoplasts might be the only means of testing *Pst* effectors for being avirulence factors for any cognate YR gene. A cell death detection assay is developed on wheat protoplasts. The protoplasts isolated can be used from a wheat line having a particular R gene by expressing effectors of a race with known virulence and avirulence pathogenicity possessing a cognate Avr to that of the R-gene. The method detects luciferase activity (Saur et al., 2019); it demonstrates the detection of significant loss of luciferase activity due to cell death by Avr sensed YR-protein, where the mesophyll protoplasts are transfected with the luciferase, a candidate Avr, and the YR-gene. However, the method can be very cumbersome for testing hundreds of effectors on many wheat lines with different YR genes. Thus, there is an urgent need for high-throughput transient gene expression methods in intact wheat plants. In our opinion, it appears that the best possible candidate approach could be virus-mediated overexpression of genes in wheat until the time a particular strain of *A. tumefaciens* is engineered for efficient transient gene expression. Most recently, BSMV-mediated VOX was successfully applied to express the predicted effector proteins from *Pst* to identify the *AvrSr27* gene (Upadhyaya et al., 2021). In another study, the infiltration of purified *AvrSr35*, expressed with the intact signal peptide in *Escherichia coli*, into the wheat with *Sr35* resistance gene resulted in an HR. The method is useful for determining Avr proteins in the true host (Salcedo et al., 2017). However, the applicability of this approach for high-throughput screening of effectors for seeking out Avrs can be cumbersome. In our opinion, the search for *Avr* genes of *Pst* requires genetic studies on isolates of the races, as it has been proven successful for finding *Avr* genes of wheat stem rust pathogen. The precisely identified virulence and avirulence of the *Pst* isolates of the same race can be utilized for comparing the genome and transcriptome sequences. Searching for natural mutations resulting in different virulence/avirulence by comparing the sequences leads to the identification of the candidate genes. For *Pst*, it is now possible to generate a segregating population of the isolates by fertilization, since its sexual reproduction is possible in the alternate host, which is determined to be barberry. On the segregating population, a high-density genetic map can be generated using single-nucleotide markers, determined by sequencing. Indeed, such a study produced candidate *PstAvr* genes by comparing the sequences of ethyl-methane sulfonate (EMS)-generated mutants with the progenitor isolate (Xia et al., 2020). Thus, the goal of identifying *Avr* genes of *Pst* is now within reach.

Thordal-Christensen proposed a model, known as the “iceberg model” as a view on ETI. In the model, it is pointed out that most of the NLRs, the effectors, and the effector targets keep one another in a silent state. The model helps explain the existence of many NLRs, effectors, and lesion mutants, also why many effectors appear to enhance virulence due to suppression of PTI (as in **Table 3**). It is argued that many of these effectors indeed cause effector-triggered susceptibility; when silenced, contributing to virulence indirectly, they may be misinterpreted as suppressors of PTI. In this model, it is claimed that what is observed most often is the tip of the iceberg (Thordal-Christensen, 2020). This view questions the presence of numerous NLRs and emphasizes that few are R-genes, and a few effectors are cognate Avrs. Finding the primary components of ETI (R/Avr) may be relatively easier than a full understanding of what is happening below the surface of the iceberg in plant immunity that requires time and huge effort.

## Author contributions

MA, NW, and AO conceived the idea and wrote the manuscript. NW, YC, WJ, XZ, XRZ, YG and ZX gathered most of the literature regarding the topic. NW, YC, and WJ participated in the classification of the selected papers in informative tables. MA and YC prepared the figure. MA and NW revised the manuscript. All authors contributed to the article and approved the submitted version.

## Funding

This work was supported by a grant from Dalian University of Technology (DUT18RC(3)062).

## Conflict of interest

The authors declare that the research was conducted in the absence of any commercial or financial relationships that could be construed as a potential conflict of interest.

## Publisher’s note

All claims expressed in this article are solely those of the authors and do not necessarily represent those of their affiliated organizations, or those of the publisher, the editors and the reviewers. Any product that may be evaluated in this article, or claim that may be made by its manufacturer, is not guaranteed or endorsed by the publisher.
