# Supplementary material for: Puccinia striiformis f. sp. tritici effectors in wheat immune responses
Source: Front Plant Sci. 2022 Nov 7;13:1012216. doi: 10.3389/fpls.2022.1012216 (PMC9677129; doi:10.3389/fpls.2022.1012216)
Supplement: Supplementary file 1 [file Table_1.docx]

**Supplementary Table 1**. Databases and prediction tools in use for effector gene mining.

| **Name** | **Description** | **URL** | **References** |
| --- | --- | --- | --- |
| **Fungal gene prediction and annotation** | | | |
| Blast2GO | A platform for functional annotation of genomic datasets. | www.blast2go.com/ | Conesa et al., 2005 |
| Evidence  Modeller | EVM integrates different prediction results into a consensus gene set. | evidencemodeler.github.io | Haas et al., 2008 |
| Galaxy | A platform designed with a user-friendly interface for researchers, could be used for effector mining. | usegalaxy.org/ | Blankenberg et al., 2010 |
| TransDecoder | Predict open reading frame from transcript sequences. | github.com/TransDecoder/TransDecoder/wiki | Haas et al., 2013 |
| SnowyOwl | An analysis pipeline customized to fungal gene prediction. | snowyowl.sourceforge.io | Reid et al., 2014 |
| CodingQuarry | Gene predictor in fungal genomes to capture most effector genes. | codingquarry.sourceforge.io | Testa et al., 2015 |
| BlastKOALA | A KEGG’s tool for annotating genome and metagenome sequences. | www.kegg.jp/blastkoala | Kanehisa et al., 2016 |
| AmiGO 2 | Gene ontology and annotation database. | amigo.geneontology.org/amigo | Carbon and Mungall, 2018 |
| BRAKER2 | An automatic protein coding gene prediction for fungal genome. | github.com/Gaius-Augustus/BRAKER | Brůna et al., 2021 |
| DESeq | Differential gene expression analysis based on the negative binomial distribution. Effectors are characterized by high expression in infection/stage specific/haustoria expressed. | bioconductor.org/packages/DESeq2 | 10.18129/B9.bioc.DESeq2 |
| **Analysis of homologs or orthologs** | | | |
| PSI-BLAST | Provide a means of detecting distant relationships between proteins. | ncbi.nlm.nih.gov/BLAST | Altschul et al., 1997 |
| HHblits | Detect protein sequence homologues via HMM-HMM alignment. | github.com/soedinglab/hh-suite | Remmert et al., 2012 |
| NCBI-BLAST | General sequence identification and similarity searches. | blast.ncbi.nlm.nih.gov/Blast.cgi | Boratyn et al., 2013 |
| HMMER | Search sequence databases for homologs and make alignments. | www.ebi.ac.uk/Tools/hmmer | Potter et al., 2018 |
| Orthovenn2 | Identify and compare orthologous genes clusters from multiple species. | orthovenn2.bioinfotoolkits.net | Xu et al., 2019 |
| EffectorDB | Database of predicted rare orthologous groups or lateral gene transfer groups in fungal effectors. | effectordb.com |  |
| **Analysis of conserved motif, domain, and protein family** | | | |
| TribeMCL | A method for clustering proteins into protein families. | metacpan.org/pod/Bio::Tools::Run::TribeMCL | Enright et al., 2003 |
| MEME Suite | Motif discovery, enrichment, and analysis tools. | meme-suite.org | Bailey et al., 2015 |
| NCBI-CDD | ‘Conserved Domain Database’ and domain analysis tool. | ncbi.nlm.nih.gov/cdd | Lu et al., 2020 |
| InterPro | Classify proteins into families and predict domains. | www.ebi.ac.uk/interpro | Blum et al., 2021 |
| Pfam 35.0 | Database for finding conserved and annotated domains in proteins. | pfam.xfam.org | Mistry et al., 2021 |
| SMART | A tool for detecting protein domains and domain architectures. | smart.embl.de | Letunic et al., 2021 |
| **Genomic analysis** | | | |
| MUMmer | General purpose pairwise genome alignment.  Useful for finding presence absence variations. | mummer.sourceforge.net | Kurtz et al., 2004 |
| PAML 4 | Maximum likelihood methods for detection of diversifying selection via CODEML. | abacus.gene.ucl.ac.uk/software/paml.html | Yang, 2007 |
| RIPCAL | Software for calculating repeat-induced point mutation in fungal genes. | sourceforge.net/projects/ripcal | Hane and Oliver, 2008 |
| SnpEff | General SNP annotation and methods for predicting SNP effects. | pcingola.github.io/SnpEff | Cingolani et al., 2012 |
| Progressive  Cactus | Multiple genome alignment appropriate for eukaryotic genomes.  Useful for finding presence absence variations. | github.com/glennhickey/progressiveCactus | Nguyen et al., 2014 |
| Occultercut | Detection of AT-rich regions in genomes, which may contain effectors. | sourceforge.net/projects/occultercut | Testa et al., 2016 |
| **Prediction of effector-like protein properties** | | | |
| LED | A database about lipases and lipase associated proteins. | www.led.uni-stuttgart.de | Fischer and Pleiss, 2003 |
| PredGPi | A Glycosylphosphatidylinositol (GPI) anchor predictor for fungal proteins. | gpcr.biocomp.unibo.it/predgpi/pred.htm | Pierleoni et al., 2008 |
| DFVF | Database of virulence factors in fungal pathogens. | sysbio.unl.edu/DFVF | Lu et al., 2012 |
| FungiDB | An integrated bioinformatics resource for fungi. | FungiDB.org | Stajich et al., 2012 |
| fPoxDB | ‘Fungal peroxidase database’ fabricated via a prediction on fungal genomes. | peroxidase.riceblast.snu.ac.kr | Choi et al., 2014 |
| dbCAN2 | A web of ‘Carbohydrate-active enzyme’ (CAZymes) for automated annotation. | bcb.unl.edu/dbCAN2 | Zhang et al., 2018 |
| MEROPS | Database for peptidases, inhibitors, and substrates of peptidases. | www.ebi.ac.uk/merops | Rawlings et al., 2018 |
| NetSurfP 2.0 | Predict the surface accessibility and secondary structure of protein sequence. | www.cbs.dtu.dk/services/NetSurfP | Klausen et al., 2019 |
| PSIPRED | Secondary structure prediction and various analysis. | bioinf.cs.ucl.ac.uk/psipred | Buchan and Jones, 2019 |
| PHI-base | ‘Pathogen Host Interaction database’ provides information about experimentally tested pathogenicity, virulence and effector genes of pathogens infecting various host organisms. | phi-base.org | Urban et al., 2020 |
| EffectorP 3.0 | Fungal effector prediction via machine learning method. | effectorp.csiro.au | Sperschneider et al., 2021 |
| NetGPI 1.1 | NetGPI is a prediction tool of GPI-anchoring or glypiation. | github.com/mhgislason/netgpi-1.1 | Gíslason et al., 2021 |
| CAZy | CAZymes database of enzymes that degrade, modify, or create glycosidic bonds. | www.cazy.org | Drula et al., 2022 |
| **Secretome analysis** | | | |
| SPScan | SPScan scans protein sequences for the presence of secretory signal peptides. | rothlab.ucdavis.edu/genhelp/spscan.html | Nielsen, et al., 1997 |
| PrediSi | An online software for the prediction of signal peptides. | predisi.de | Hiller et al., 2004 |
| SecretomeP | Ab initio prediction of non-classical protein secretion. | www.cbs.dtu.dk/services/SecretomeP | Bendtsen et al., 2005 |
| Signal-CF | A method for predicting protein signal peptide and its cleavage site. | www.csbio.sjtu.edu.cn/bioinf/Signal-CF | Chou and Shen, 2007 |
| FSD | A platform for studying fungal secretome using the data of stored fungal species. | fsd.snu.ac.kr | Choi et al., 2010 |
| FunSecKB | A database contains predicted fungal secreted proteins from NCBI RefSeq. | proteomics.ysu.edu/secretomes | Lum and Min, 2011 |
| Secretool | Prediction and characterization of fungal secreted proteins. | genomics.cicbiogune.es/SECRETOOL | Cortázar et al., 2014 |
| TOPCONS | Prediction of membrane protein topology and signal peptides. | octopus.cbr.su.se | Tsirigos et al., 2015 |
| SignalP 6.0 | N-terminus secretion signal prediction based on artificial neural networks and protein language models. A common first step in effector prediction. | www.cbs.dtu.dk/services/SignalP | Teufel et al., 2022 |
| **Prediction for removing secreted proteins with transmembrane domains** | | | |
| PRED-TMR2 | A system for the classification of transmembrane proteins. | athina.biol.uoa.gr/PRED-TMR2/input.html | Pasquier and Hamodrakas, 1999 |
| TMHMM 2.0 | Prediction of transmembrane helices of proteins. | www.cbs.dtu.dk/services/TMHMM | Krogh et al., 2001 |
| MINNOU | Sequence-based prediction of relative lipid accessibility. | minnou.cchmc.org | Cao et al., 2006 |
| Phobius | A combined transmembrane topology and signal peptide predictor. | phobius.sbc.su.se | Käll et al., 2007 |
| SOMRuler | A novel interpretable transmembrane helices predictor. | www.csbio.sjtu.edu.cn/bioinf/SOMRuler | Yu et al., 2011 |
| MemBrain | A web server developed for transmembrane protein structure prediction. | www.csbio.sjtu.edu.cn/bioinf/MemBrain | Feng et al., 2020 |
| **Subcellular localization prediction** | | | |
| ProtComp 9.0 | Predict the subcellular localization for fungi and plant proteins. | linux1.softberry.com |  |
| Cello | Prediction of subcellular localization of secretory proteins. | cello.life.nctu.edu.tw | Yu et al., 2006 |
| WoLF PSORT | Prediction of subcellular localization of proteins. | wolfpsort.hgc.jp | Horton et al., 2007 |
| cNLS Mapper | Prediction of nuclear localization signals in proteins. | nls-mapper.iab.keio.ac.jp | Kosugi et al., 2009 |
| YLoc | A prediction system for protein subcellular localization prediction. | github.com/KohlbacherLab/YLoc | Briesemeister et al., 2010 |
| Cello2GO | Prediction of subcellular localization of proteins with annotation. | cello.life.nctu.edu.tw/cello2go | Yu et al., 2014 |
| LOCALIZER | Subcellular localization prediction of plant and effector proteins. | localizer.csiro.au | Sperschneider et al., 2017 |
| ApoplastP | Prediction of proteins localized into apoplast region of plant. | apoplastp.csiro.au | Sperschneider et al., 2018 |
| TargetP 2.0 | Prediction of subcellular or extracellular localization of proteins. | www.cbs.dtu.dk/services/TargetP | Almagro Armenteros et al., 2019 |

**References**

Almagro Armenteros, J. J., Salvatore, M., Emanuelsson, O., Winther, O., Von Heijne, G., Elofsson, A., et al. (2019). Detecting sequence signals in targeting peptides using deep learning. *Life Sci. Alliance* 2:e201900429. doi: 10.26508/lsa.201900429

Altschul, S. F., Madden, T. L., Schäffer, A. A., Zhang, J., Zhang, Z., Miller, W., et al. (1997). Gapped BLAST and PSI-BLAST: a new generation of protein database search programs. *Nucleic Acids Res.* 25, 3389–3402. doi: 10.1093/nar/25.17.3389

Bailey, T. L., Johnson, J., Grant, C. E., and Noble, W. S. (2015). The MEME Suite. *Nucleic Acids Res.* 43, W39–W49. doi: 10.1093/nar/gkv416

Bendtsen, J. D., Kiemer, L., Fausbøll, A., and Brunak, S. (2005). Non-classical protein secretion in bacteria. *BMC Microbiol.* 5:58. doi: 10.1186/1471-2180-5-58

Blankenberg, D., Von Kuster, G., Coraor, N., Ananda, G., Lazarus, R., Mangan, M., et al. (2010). Galaxy, a web-based genome analysis tool for experimentalists. *Curr. Protoc. Mol. Biol.* 19:Unit 19.1021. doi: 10.1002/0471142727.mb1910s89

Blum, M., Chang, H. Y., Chuguransky, S., Grego, T., Kandasaamy, S., Mitchell, A., et al. (2021). The InterPro protein families and domains database: 20 years on. *Nucleic Acids Res.* 49, D344–D354. doi: 10.1093/nar/gkaa977

Boratyn, G. M., Camacho, C., Cooper, P. S., Coulouris, G., Fong, A., Ma, N., et al. (2013). BLAST: a more efficient report with usability improvements. *Nucleic Acids Res.* 41, W29–W33. doi: 10.1093/nar/gkt282

Briesemeister, S., Rahnenführer, J., and Kohlbacher, O. (2010). YLoc – an interpretable web server for predicting subcellular localization. *Nucleic Acids Res.* 38, W497–W502. doi: 10.1093/nar/gkq477

Brůna, T., Hoff, K. J., Lomsadze, A., Stanke, M., and Borodovsky, M. (2021). BRAKER2: automatic eukaryotic genome annotation with GeneMark-EP+ and AUGUSTUS supported by a protein database. *NAR Genom. Bioinform.* 3:1. doi:10.1093/nargab/lqaa108

Buchan, D. W. A., and Jones, D. T. (2019). The PSIPRED protein analysis workbench: 20 years on. *Nucleic Acids Res.* 47, W402–W407. doi: 10.1093/nar/gkz297

Cao, B., Porollo, A., Adamczak, R., Jarrell, M., and Meller, J. (2006). Enhanced recognition of protein transmembrane domains with prediction-based structural profiles. *Bioinformatics* 22, 303–309. doi: 10.1093/bioinformatics/bti784

Carbon, S., and Mungall, C. (2018). Gene Ontology Data Archive (2021-12-15) [Data set]. Zenodo. doi: 10.5281/zenodo.5789900

Choi, J., Détry, N., Kim, K. T., Asiegbu, F. O., Valkonen, J. P., and Lee, Y. H. (2014). fPoxDB: fungal peroxidase database for comparative genomics. *BMC Microbiol.* 14:117. doi: 10.1186/1471-2180-14-117

Choi, J., Park, J., Kim, D., Jung, K., Kang, S., and Lee, Y. H. (2010). Fungal Secretome Database: Integrated platform for annotation of fungal secretomes. *BMC Genomics* 11:105 doi: 10.1186/1471-2164-11-105

Chou, K. C., and Shen, H. B. (2007). Signal-CF: A subsite-coupled and window-fusing approach for predicting signal peptides. *Biochem. Biophys. Res. Commun.* 357, 633–640. doi: 10.1016/j.bbrc.2007.03.162

Cingolani, P., Platts, A., Wang, L.L., Coon, M., Nguyen, T., Wang, L., et al. (2012). A program for annotating and predicting the effects of single nucleotide polymorphisms, SnpEff: SNPs in the genome of *Drosophila melanogaster* strain w^1118^; iso-2; iso-3. *Fly* 6:80–92. doi: 10.4161/fly.19695

Conesa, A., Götz, S., García-Gómez, J.M., Terol, J., Talón, M., and Robles, M. (2005). Blast2GO: A universal tool for annotation, visualization and analysis in functional genomics research. *Bioinformatics* 21, 3674–3676. doi: 10.1093/bioinformatics/bti610

Cortázar, A. R., Aransay, A. M., Alfaro, M., Oguiza, J. A., and Lavín, J. L. (2014). SECRETOOL: Integrated secretome analysis tool for fungi. *Amino Acids* 46, 471–473. doi: 10.1007/s00726-013-1649-z

Drula, E., Garron, M. L., Dogan, S., Lombard, V., Henrissat, B., and Terrapon, N. (2022). The carbohydrate-active enzyme database : functions and literature. *Nucleic Acids Res.* 50, D571–D577. doi: 10.1093/nar/gkab1045

Enright, A. J., Kunin, V., and Ouzounis, C. A. (2003). Protein families and TRIBES in genome sequence space. *Nucleic Acids Res.* 31, 4632–4638. doi: 10.1093/nar/gkg495

Feng, S. H., Zhang, W. X., Yang, J., Yang, Y., and Shen, H. B. (2020). Topology prediction improvement of α-helical transmembrane proteins through helix-tail modeling and multiscale deep learning fusion. *J. Mol. Biol.* 432, 1279–1296. doi: 10.1016/j.jmb.2019.12.007

Fischer, M., and Pleiss, J. (2003). The Lipase Engineering Database: a navigation and analysis tool for protein families. *Nucleic Acids Res.* 31, 319–321. doi: 10.1093/nar/gkg015

Gíslason, M. H., Nielsen, H., Almagro Armenteros, J. J., and Johansen, A. R. (2021). Prediction of GPI-anchored proteins with pointer neural networks. *Curr. Res. Biotech.* 3, 6–13. doi: 10.1016/j.crbiot.2021.01.001

Haas, B. J., Papanicolaou, A., Yassour, M., Grabherr, M., Blood, P. D., Bowden, J., et al. (2013). *De novo* transcript sequence recostruction from RNA-Seq: reference generation and analysis with Trinity. *Nat. Protoc.* 8:8. doi:10.1038/nprot.2013.084

Haas, B. J., Salzberg, S. L., Zhu, W., Pertea, M., Allen, J. E., Orvis, J., et al. (2008). Automated eukaryotic gene structure annotation using EVidenceModeler and the Program to Assemble Spliced Alignments. *Genome Biology* 9:R7. doi: 10.1186/gb-2008-9-1-r7

Hane, J. K., and Oliver, R. P. (2008). RIPCAL: a tool for alignment-based analysis of repeat-induced point mutations in fungal genomic sequences. *BMC Bioinformatics* 9:478. doi: 10.1186/1471-2105-9-478

Hiller, K., Grote, A., Scheer, M., Münch, R., and Jahn, D. (2004). PrediSi: prediction of signal peptides and their cleavage positions. *Nucleic Acids Res.* 32, W375–W379. doi: 10.1093/nar/gkh378

Horton, P., Park, K. J., Obayashi, T., Fujita, N., Harada, H., Adams-Collier, C. J., et al. (2007). WoLF PSORT: protein localization predictor. *Nucleic Acids Res.* 35, W585–W587. doi: 10.1093/nar/gkm259

Käll, L., Krogh, A., and Sonnhammer, E. L. L. (2007). Advantages of combined transmembrane topology and signal peptide prediction – the Phobius web server. *Nucleic Acids Res.* 35, W429–W432. doi: 10.1093/nar/gkm256

Kanehisa, M., Sato, Y., and Morishima, K. (2016). BlastKOALA and GhostKOALA: KEGG tools for functional characterization of genome and metagenome sequences. *J. Mol. Biol.* 428, 726–731. doi: 10.1016/j.jmb.2015.11.006

Klausen, M. S., Jespersen, M. C., Nielsen, H., Jensen, K. K., Jurtz, V. I., Sønderby, C. K., et al. (2019). NetSurfP-2.0: Improved prediction of protein structural features by integrated deep learning. *Proteins* 87, 520–527. doi: 10.1002/prot.25674

Kosugi, S., Hasebe, M., Tomita, M., and Yanagawa, H. (2009). Systematic identification of cell cycle-dependent yeast nucleocytoplasmic shuttling proteins by prediction of composite motifs. *Proc. Natl. Acad. Sci. U.S.A.* 106, 10171–10176. doi: 10.1073/pnas.0900604106

Krogh, A., Larsson, B., von Heijne, G., and Sonnhammer, E. L. L. (2001). Predicting transmembrane protein topology with a hidden Markov model: application to complete genomes. *J. Mol. Biol.* 305, 567–580. doi: 10.1006/jmbi.2000.4315

Kurtz, S., Phillippy, A., Delcher, A. L., Smoot, M., Shumway, M., Antonescu, C., et al. (2004). Versatile and open software for comparing large genomes. *Genome Biol.* 5:R12. doi: 10.1186/gb-2004-5-2-r12

Letunic, I., Khedkar, S., and Bork, P. (2021). SMART: recent updates, new developments and status in 2020. *Nucleic Acids Res.* 49, D458–D460. doi: 10.1093/nar/gkaa937

Lu, S., Wang, J., Chitsaz, F., Derbyshire, M. K., Geer, R. C., Gonzales, N. R., et al. (2020). CDD/SPARCLE: the conserved domain database in 2020. *Nucleic Acids Res.* 48, D265–D268. doi: 10.1093/nar/gkz991

Lu, T., Yao, B., and Zhang, C. (2012). DFVF: database of fungal virulence factors. *Database* 2012:bas032. doi: 10.1093/database/bas032

Lum, G., and Min, X. J. (2011). FunSecKB: the Fungal Secretome KnowledgeBase. 2011:bar001 doi: 10.1093/database/bar001

Mistry, J., Chuguransky, S., Williams, L., Qureshi, M., Salazar, G. A., Sonnhammer, E. L. L., et al. (2021). Pfam: The protein families database in 2021. *Nucleic Acids Res.* 49, D412–D419. doi: 10.1093/nar/gkaa913

Nguyen, N., Hickey, G., Raney, B. J., Armstrong, J., Clawson, H., Zweig, A., et al. (2014). Comparative assembly hubs: Web-accessible browsers for comparative genomics. *Bioinformatics* 30, 3293–3301. doi: 10.1093/bioinformatics/btu534

Nielsen, H., Engelbrecht, J., Brunak, S., von Heijne, G. (1997) Identification of prokaryotic and eukaryotic signal peptides and prediction of their cleavage sites. *Protein Eng. Des. Sel.* 10, 1–6. doi: 10.1093/protein/10.1.1

Pasquier, C., and Hamodrakas, S. J. (1999). An hierarchical artificial neural network system for the classification of transmembrane proteins. *Protein Eng.* 12, 631–634. doi: 10.1093/bioinformatics/btu534

Pierleoni, A., Martelli, P. L., and Casadio, R. (2008). PredGPI: a GPI-anchor predictor. *BMC Bioinformatics* 9:392. doi: 10.1186/1471-2105-9-392

Potter, S. C., Luciani, A., Eddy, S. R., Park, Y., Lopez, R., and Finn, R. D. (2018). HMMER web server: 2018 update. *Nucleic Acids Res.* 46, W200–W204. doi: 10.1093/nar/gky448

Rawlings, N. D., Barrett, A. J., Thomas, P. D., Huang, X., Bateman, A., and Finn, R. D. (2018). The *MEROPS* database of proteolytic enzymes, their substrates and inhibitors in 2017 and a comparison with peptidases in the PANTHER database. *Nucleic Acids Res.* 46, D624–D632. doi: 10.1093/nar/gkx1134

Reid, I., O’Toole, N., Zabaneh, O., Nourzadeh, R., Dahdouli, M., Abdellateef, M., et al. (2014). SnowyOwl: accurate prediction of fungal genes by using RNA-Seq and homology information to select among ab initio models. *BMC Bioinformatics* 15:229. doi: 10.1186/1471-2105-15-229

Remmert, M., Biegert, A., Hauser, A., and Söding, J. (2012). HHblits: lightning-fast iterative protein sequence searching by HMM-HMM alignment. *Nat. Methods* 9, 173–175. doi: 10.1038/nmeth.1818

Sperschneider, J., and Dodds, P. (2021). EffectorP 3.0: prediction of apoplastic and cytoplasmic effectors in fungi and oomycetes. *Mol. Plant Microbe Interact.* doi: 10.1094/mpmi-08-21-0201-r

Sperschneider, J., Catanzariti, A. M., DeBoer, K., Petre, B., Gardiner, D. M., Singh, K. B., et al. (2017). LOCALIZER: subcellular localization prediction of both plant and effector proteins in the plant cell. *Sci. Rep.* 7:44598 doi: 10.1038/srep44598

Sperschneider, J., Dodds, P. N., Singh, K. B., and Taylor, J. M. (2018). ApoplastP: prediction of effectors and plant proteins in the apoplast using machine learning. *New Phytol.* 217, 1764–1778. doi: 10.1111/nph.14946

Stajich, J. E., Harris, T., Brunk, B. P., Brestelli, J., Fischer, S., Harb, O. S., et al. (2012). FungiDB : an integrated functional genomics database for fungi. 40, D675–D681. doi: 10.1093/nar/gkr918

Testa, A. C., Hane, J. K., Ellwood, S. R., and Oliver, R. P. (2015). CodingQuarry: highly accurate hidden Markov model gene prediction in fungal genomes using RNA-seq transcripts. *BMC Genomics* 16:170. doi: 10.1186/s12864-015-1344-4

Testa, A. C., Oliver, R. P., and Hane, J. K. (2016). OcculterCut: A comprehensive survey of AT-rich regions in fungal genomes. *Genome Biol. Evol.* 8, 2044–2064. doi: 10.1093/gbe/evw121

Teufel, F., Almagro Armenteros, J. J., Johansen, A. R., Gíslason, M. H., Pihl, S. I., Tsirigos, K. D., et al. (2022). SignalP 6.0 predicts all five types of signal peptides using protein language models. *Nat. Biotechnol.* doi: 10.1038/s41587-021-01156-3

Tsirigos, K. D., Peters, C., Shu, N., Käll, L., and Elofsson, A. (2015). The TOPCONS web server for consensus prediction of membrane protein topology and signal peptides. *Nucleic Acids Res.* 43, W401–W407. doi: 10.1093/nar/gkv485

Urban, M., Cuzick, A., Seager, J., Wood, V., Rutherford, K., Venkatesh, S. Y., et al. (2020). PHI-base: the pathogen-host interactions database. *Nucleic Acids Res.* 48, D613–D620. doi: 10.1093/nar/gkz904

Xu, L., Dong, Z., Fang, L., Luo, Y., Wei, Z., Guo, H., et al. (2019). OrthoVenn2: A web server for whole-genome comparison and annotation of orthologous clusters across multiple species. *Nucleic Acids Res.* 47, W52–W58. doi: 10.1093/nar/gkz333

Yang, Z. (2007). PAML 4: Phylogenetic analysis by maximum likelihood. *Mol. Biol. Evol.* 24, 1586–1591. doi: 10.1093/molbev/msm088

Yu, C. S., Chen, Y. C., Lu, C. H., Hwang, J. K. (2006). Prediction of protein subcellular localization. *Proteins Struct. Funct. Bioinf.* 64, 643–651. doi: 10.1007/978-1-0716-1641-3_12

Yu, C. S., Cheng, C. W., Su, W. C., Chang, K. C., Huang, S. W., Hwang, J. K., et al. (2014). CELLO2GO: A web server for protein subCELlular LOcalization prediction with functional gene ontology annotation. *PLoS ONE* 9:e99368. doi:10.1371/journal.pone.0099368

Yu, D., Shen, H., and Yang, J. (2011). SOMRuler: A novel interpretable transmembrane helices predictor. *IEEE Trans Nanobioscience* 10, 121–129. doi: 10.1109/TNB.2011.2160730

Zhang, H., Yohe, T., Huang, L., Entwistle, S., Wu, P., Yang, Z., et al. (2018). dbCAN2: a meta server for automated carbohydrate-active enzyme annotation. *Nucleic Acids Res.* 46, W95–W101. doi: 10.1093/nar/gky418
